# Antioxidant Effects and Probiotic Properties of *Latilactobacillus sakei* MS103 Isolated from Sweet Pickled Garlic

**DOI:** 10.3390/foods12234276

**Published:** 2023-11-27

**Authors:** Heng Li, Changlin Chen, Yuanxin Li, Zhengqiang Li, Chen Li, Chang Luan

**Affiliations:** 1 College of Information Technology, Jilin Agricultural University, Chuangchun 130118, China; hengl@jlau.edu.cn (H.L.); 20231321@mails.jlau.edu.cn (C.C.); 2 Key Laboratory for Molecular Enzymology and Engineering of the Ministry of Education, College of Life Sciences, Jilin University, Changchun 130012, China; yuanxin22@mails.jlu.edu.cn (Y.L.); lzq@jlu.edu.cn (Z.L.); 3 State Key Laboratory for Zoonotic Diseases, Key Laboratory for Zoonosis Research of the Ministry of Education, Institute of Zoonosis, College of Veterinary Medicine, Jilin University, Changchun 130062, China; 4 State Key Laboratory of Black Soils Conservation and Utilization, Northeast Institute of Geography and Agroecology, Chinese Academy of Sciences, Changchun 130102, China

**Keywords:** *Latilactobacillus sakei* MS103, probiotic, antioxidant, H_2_O_2_, *P. gingivalis*

## Abstract

Fermented vegetable-based foods, renowned for their unique flavors and human health benefits, contain probiotic organisms with reported in vitro antioxidative effects. This study investigates the probiotic properties of *Latilactobacillus sakei* MS103 (*L. sakei* MS103) and its antioxidant activities using an in vitro oxidative stress model based on the hydrogen peroxide (H_2_O_2_)-induced oxidative damage of RAW 264.7 cells. *L. sakei* MS103 exhibited tolerance to extreme conditions (bile salts, low pH, lysozyme, H_2_O_2_), antibiotic sensitivity, and auto-aggregation ability. Moreover, *L. sakei* MS103 co-aggregated with pathogenic *Porphyromonas gingivalis* cells, inhibited *P. gingivalis*-induced biofilm formation, and exhibited robust hydrophobic and electrostatic properties that enabled it to strongly bind to gingival epithelial cells and HT-29 cells for enhanced antioxidant effects. Additionally, *L. sakei* MS103 exhibited other antioxidant properties, including ion-chelating capability and the ability to effectively scavenge superoxide anion free radicals, hydroxyl, 2,2′-azino-bis (3-ethylbenzothiazoline-6-sulfonic acid, and 2,2-diphenyl-1-picrylhydrazyl. Furthermore, the addition of live or heat-killed *L. sakei* MS103 cells to H_2_O_2_-exposed RAW 264.7 cells alleviated oxidative stress, as reflected by reduced malondialdehyde levels, increased glutathione levels, and the up-regulated expression of four antioxidant-related genes (*gshR2, gshR4, Gpx,* and *npx*). These findings highlight *L. sakei* MS103 as a potential probiotic capable of inhibiting activities of *P. gingivalis* pathogenic bacteria and mitigating oxidative stress.

## 1. Introduction

Fermentation, a time-honored method of food preservation, profoundly influences both nutritional quality and flavor [1]. Across the globe, this technique is widely embraced to enhance the taste and maintain the nutritional value of vegetables. China, renowned for its culinary diversity, produces iconic products, such as Sichuan pickles and Suancai, that are crafted using unique regional methods. Various cultures have developed their own traditional vegetable pickles. Korea’s celebrated kimchi, for instance, involves the salting of cabbages and radishes, rinsing to remove excess salt, and fermentation of the vegetables in kimchi paste, while Nepal’s gundruk is fermented without added salt [2]. Turkey’s tursu is a beloved traditional pickle created by submerging vegetables in brine and allowing them to ferment at room temperature, which is a similar method to that used to prepare Chinese pickles (paocai).

Garlic, a global natural spice and ingredient of fermented foods, has been extensively studied for its diverse health benefits. These attributes include antimicrobial and anticancer properties, immune system-enhancing effects, antioxidant effects, and various other beneficial activities [3] that have stimulated the development of a broad array of garlic-based products, such as garlic oil, flakes, salt, and paste. Moreover, the addition of garlic during the pickling process not only prevents the growth of unwanted microorganisms but also encourages the proliferation of beneficial *Lactiplantibacillus* species [4]. In northeastern China, sweet pickled garlic, with its rich contents of protein, healthy fats, sugar, and other essential nutrients, is considered a wholesome delicacy [4,5,6,7,8]. The sweet pickled garlic is always eaten directly, which may be beneficial for preventing oral disease.

The Food and Agriculture Organization of the United Nations (FAO)/World Health Organization (WHO) defined probiotics as “live microorganisms that, when administered in adequate amounts, confer a health benefit on the host”. Lactic acid bacteria (LAB), a prominent group of probiotic organisms [2], were initially isolated from milk and have since been detected in many fermented products with ancient origins [9]. Due to their longstanding role in food preservation, LAB are currently viewed as generally recognized as safe (GRAS) when used for this purpose. Furthermore, LAB possess essential characteristics associated with enhanced food safety and shelf life. These benefits have been linked to their abilities to inhibit pathogen growth during antimicrobial compounds production, including diacetyl, hydrogen peroxide and others. LAB also compete with pathogens for nutrients, further contributing to food safety [10,11].

LAB and its fermentates provide health benefits in several studies. For example, Fasano and Budelli reported that LAB fermentates improved both gut health and overall health, while studies conducted by a group of Food for Health Ireland (FHI) researchers revealed that the consumption of LAB fermentates of reconstituted skim milk (RSM) curbed weight gain by regulating appetite. In an independent study, Casey and colleagues demonstrated that using a mixture of five LAB strains isolated from milk fermentation on pigs infected with *Salmonella* serovar Typhimurium resulted in a significant reduction in *Salmonella* counts in the pigs’ feces, contributing to the overall enhanced health of the animals. Furthermore, LAB fermentates have been shown to mitigate the effects of food-borne pathogens, as reported by Morgan and colleagues [12].

It is worth noting that the health impacts of LAB also extend to oral health, where they may exert harmful effects by contributing to the development of oral disorders, such as dental caries and periodontitis [13]. *P. gingivalis* is a common pathogen, which was considered associated with periodontal diseases [14]. Given the multifaceted benefits of LAB, understanding their specific characteristics and functions is essential when exploring their various potential applications in promoting health—especially, the LAB effect on the *P. gingivalis* with biofilm formation.

Oxidative stress, driven by reactive oxygen species (ROS) overproduction in oxidative chemical processes, can irreversibly damage cells and tissues. Several diseases including heart disease, cancer and so on were closely associated with ROS damage phenomena [15,16,17]. Recent evidence from in vivo and in vitro studies has demonstrated significant antioxidant capabilities of probiotic bacteria linked to valuable health benefits. In particular, certain strains of *lactobacilli* and *bifidobacteria* have gained recognition for their impressive antioxidative properties, which is a crucial factor in strengthening the body’s antioxidant defenses and mitigating oxidative stress [18,19].

Within the gut environment, probiotic bacteria exert their antioxidant effects by releasing antioxidant molecules, secreting antioxidant enzymes, directly scavenging ROS, and chelating iron to prevent iron-catalyzed oxidation [20,21]. As a result, the use of probiotics with their antioxidant abilities holds promise as a reliable approach to mitigating the damage caused by oxidative stress.

*Latilactobacillus sakei*, a Gram-positive lactic acid bacillus, was initially isolated from Japanese sake in 1934 by Katagiti et al. It has since been found in other foods, including meat products and various plant-derived foods, such as fermented cabbage. In particular, *L. sakei* isolated from kimchi has been associated with various potential health-related benefits, including the alleviation of obesity, inflammatory bowel disease, and atopic dermatitis [22].

In 2021, genomic information was obtained for 56 diverse *L. sakei* strains, revealing several LAB genes associated with antioxidative capabilities and potential health benefits [23,24]. In our preliminary study, we demonstrated that *L. sakei* MS103 isolated from a traditional Chinese fermented food (sweet pickle garlic) possessed potent abilities in scavenging 2,2-diphenyl-1-picrylhydrazyl (DPPH) and 2,2’-azino-bis (3-ethylbenzothiazoline-6-sulfonic acid (ABTS) radicals (Table S1). This finding led us to speculate that *L. sakei* MS103 may possess antioxidative properties capable of counteracting the harmful effects of pathogenic bacteria.

Here, we investigated the potential probiotic properties of *L. sakei* MS103 in vitro, including environmental tolerance, surface characteristics, antioxidative activities, antibiotic resistance, and beneficial effects on *P. gingivalis* biofilm formation. *L. sakei* MS103 antioxidant activities were assessed using the H_2_O_2_-Induced Oxidative Damage RAW 264.7 Cell Model. Our preliminary research results highlight the significant observed antioxidant benefits of *L. sakei* MS103 that justify its further evaluation as a potential health-enhancing ingredient for incorporation in dietary supplements, cosmetics, pharmaceuticals, and nutraceuticals.

## 2. Materials and Methods

### 2.1. Latilactobacillus sakei MS103 Strains and Sample Preparation

*Latilactobacillus sakei* MS103 (*L. sakei* MS103) was initially isolated from sweet pickled garlic, which is a traditional Chinese fermented food product. The *L. sakei* MS103 used in our experiment was deposited with the No. 21,362 at the China General Microbiological Culture Collection Center (CGMCC, Beijing, China). The de Man, Rogosa and Sharpe (MRS) broth (Hopebio Co., Qingdao, China) was used to inoculate the *L. sakei* MS103 (3%, *v*/*v*). The *L. sakei* MS103 was cultured for 16 h with 37 °C temperature in an anaerobic incubator. The anaerobic incubator was under the same conditions (85% N2, 10% H2, and 5% CO_2_) in all experiments. For cells and strains’ incubation, without special explanation, the temperature was maintained at 37 °C. After centrifuging at 1500× *g* for 10 min at a temperature of 4 °C, the *L. sakei* MS103 were collected. The phosphate-buffered saline (PBS) was used to wash the pellet three times. Before conducting any further experiments, the *L. sakei* MS103 was adjusted to 9 Log CFU/mL, 8 Log CFU/mL, or 7 Log CFU/mL with the PBS buffer. For the *L. sakei* MS103 culture medium group, the *L. sakei* MS103 was collected with medium and adjusted to the suitable concentration. For the live *L. sakei* MS103, the *L. sakei* MS103 culture medium was centrifuged for 10 min at the temperature of 4 °C (1500× *g*). Then, the bacterial supernatant was collected by removing the bacterial cell using a 0.22 M membrane filter [17].

To prepare heat-killed *L. sakei* MS103, bacterial cells were collected after culture, centrifuged, and washed with PBS, as described above, and then incubated at 100 °C for 15 min. Thereafter, the heat-killed cells were centrifuged at 4 °C for 10 min with 1500× *g*. After removing the supernatant, the cells were adjusted to 9 Log CFU/mL, 8 Log CFU/mL, or 7 Log CFU/mL in DMEM prior to use in experiments.

*Porphyromonas gingivalis* (*P. gingivalis*) (ATCC 3327), which was obtained from the CGMCC, was propagated on Columbia Blood Agar (Hopebio Co.) with the temperature of 37 °C for 48 h in an anaerobic incubator condition. After culture, *P. gingivalis* cells were resuspended in Brain–Heart Infusion (BHI) broth (3% *v*/*v*, Hopebio Co.) and incubated at the temperature of 37 °C for an additional 48 h.

From Otwo Biotech (Shenzhen, China), three kinds of cells, RAW 264.7 (mouse mononuclear macrophage-derived) cells, human gingival epithelial cells (HGE) and human colorectal adenocarcinoma-derived cells (HT-29), were obtained. DMEM (HyClone, Logan, UT, USA) was used to culture the cells with 10% (*v*/*v*) fetal bovine serum (FBS; HyClone). In DMEM culture medium,100 U/mL penicillin/streptomycin (Sigma-Aldrich, St. Louis, MO, USA) was added. All cells were cultured at the temperature of 37 °C in an incubator. Then, every other day, the fresh medium was used to replace the spent medium; on intervening days, cells were subcultured by removing spent medium from culture flasks and adding 0.25% trypsin–EDTA solution (Sigma-Aldrich), tapping the flasks to dislodge adherent cells from flask walls, and then diluting the resuspended cells 1:3 (by volume) in fresh medium and transferring cell suspensions to new flasks.

### 2.2. Physiological Functional Properties of L. sakei MS103

#### 2.2.1. *L. sakei* MS103 Tolerance to Pepsin, Bile, Lysozyme, and H_2_O_2_

*L. sakei* MS103 16 h cultures were cultured with pepsin (0.3% (*w*/*v*)) of MRS broth at pH 2.0, pH 3.0 and pH 5.0. Different concentrations with bovine bile (0.3% (*w*/*v*) and 0.5%(*w*/*v*)) and MRS broth were used. Lysozymes (100 μg/mL or 200 μg/mL) were added into the MRS broth. MRS broths with 0.08 mM H_2_O_2_ or 0.8 mM H_2_O_2_ were prepared. Then, cultures were incubated under aerobic conditions for 20 h (37 °C). During the culture period, every 2 h until 20 h, a UV-VIS spectrophotometer (UV-2700, Shimadzu, Kyoto, Japan) were used to measure the optical density (OD) values at 600 nm.

#### 2.2.2. *L. sakei* MS103 Auto-Aggregation Activity

*L. sakei* MS103 16 h cultures were centrifuged at 4 °C for10 min with 1500× *g*. The PBS at pH 7.0 was used to wash the pellet 3 times. Then, the pellet was resuspended in PBS buffer, and we adjusted the OD_600_ values to 1.0. Next, the suspension of OD_600_ values was measured every 2 h after being incubated at 37 °C until reaching 8 h [25,26].

The auto-aggregation ability of *L. sakei* MS103 was calculated as:

$$\mathrm{Auto-Aggregation}\;\mathrm{ability}\;(\%)=\left[1-A_{T}/A_{0}\times 100\right]$$

where *A_T_* denotes the OD_600_ value every 2 h until 8 h, and *A*_0_ denotes the OD_600_ value at 0 h.

#### 2.2.3. *L. sakei* MS103 Co-Aggregation Activity

*L. sakei* MS103 was cultured for 18 h, and *P. gingivalis* cells were harvested for 24 h. Then, cells were resuspended in sterile PBS at pH 7.0 after being washed three times. The *L. sakei* MS103 concentration was adjusted to 0.50 by OD_600_ values; the *P. gingivalis* concentration was adjusted to 0.60 at OD_600_. Then, 2 mL of *L. sakei* MS103 and 2 mL of *P. gingivalis* suspensions were shaken for 5 min (200 r/min). Afterwards, without shaking, the suspensions were incubated for 8 h at the temperature of 37 °C. The OD_600_ values were measured every 2 h until incubation 8 h [27]. The co-aggregation ability was calculated as follows:

$$\mathrm{Co-aggregation}\;\mathrm{ability}\;(\%)=\left[(\left(A_{x}+A_{y})/2-A_{mic}\right)/\left(A_{x}+A_{y}\right)/2\right]\times 100$$

where *A_x_* denotes OD_600_ values of *L. sakei* MS103 at 0 h, *A_y_* denotes OD_600_ values of *P. gingivalis* at 0 h, and *A_mix_* denotes the OD_600_ value of the mixture for both strains after incubation every 2 h.

#### 2.2.4. *L. sakei* MS103 Surface Hydrophobicity Characteristics

The microbial adhesion to hydrocarbons (MATH) method was used to determine the surface hydrophobicity of *L. sakei* MS103 in the hydrophobic organic solvent xylene, while surface charge characteristics were assessed using chloroform and ethyl acetate as the Lewis acid and Lewis base, respectively [28]. *L. sakei* MS103 was prepared using the abovementioned method. First, 0.1 mol/L KNO_3_ buffer at pH 6.2 was used to resuspend the strain pellet, which was adjusted to an OD_600_ absorbance value of 0.60. For the surface hydrophobicity assay, 1 mL of xylene, ethyl acetate, or chloroform was added to *L. sakei* MS103 (3 mL) suspension separately. For preincubating, the two-phase mixture was kept at room temperature (10 min) and vortexed (2 min). Then, the mixture was incubated at room temperature (30 min). Thereafter, the aqueous phase absorbance at OD_600_ was observed for use in calculating the cell surface hydrophobicity as based on the adherence of *L. sakei* MS103 to hydrocarbons as follows:

$$\mathrm{Cell}\;\mathrm{surface}\;\mathrm{hydrophobicity}\;(\%)=[1-A_{x}/A_{y}]\times 100$$

where *A_x_* and *A_y_* denote the OD_600_ value before and after the organic solvent treatment, respectively.

#### 2.2.5. Adhesion of *L. sakei* MS103 to HT-29 and HGE Cells

Both HT-29 and HGE cells were used to study the adhesion of *L. sakei* MS103 to host cells. First, 1 × 10^5^ cells/well of HT-29 and HGE cells were transferred into a tissue culture plate (12 wells). Then, with the cells adhered to the plate well and about 70–80% cell confluence, fresh high-glucose DMEM medium (high glucose) was used to wash and replace the medium in the well. The plates were incubated at a temperature of 37 °C for 24 h. Then, PBS buffer was used to wash the monolayers of cells for three times. For each well, the cell concentrations with 1 × 10^8^ CFU/mL were adjusted by adding 500 μL of *L. sakei* MS103. After incubating the plate for 2 h (37 °C), each well had PBS added and was washed gently three times, after which non-adherent bacteria were removed. Then, we added 2 mL of 1% (*v*/*v*) Triton X-100 (Sigma-Aldrich, USA) to each well and incubated them for 10 min. The adherent cells detached from the well surfaces [29]. The viable bacteria were counted by plate-counting method in wells. The *L. sakei* MS103 adhesion ability was calculated using the following equation:

$$\mathrm{Survival}\;\mathrm{rate}\;(\%)=\left[V_{x}/V_{y}\right]\times 100$$

where *V_x_* and *V_y_* denote the *L. sakei* MS103 cells of the total adherent count and the initial added number, respectively.

#### 2.2.6. Antibiotic Resistance

The disk diffusion method was used to detect the antibiotic susceptibility of *L. sakei* MS103 [30,31]. *L. sakei* MS103 with 8 Log CFU/mL for 1 mL was spread on the MRS agar-solidified plated surface evenly. Next, the plates were incubated for 48 h at the temperature of 37 °C with the antibiotic-containing disks placed. Vernier calipers (Aladdin Biochemical Technology Ltd., Shanghai, China; minimum resolution = 0.02 mm) were used to measure the diameter for the inhibition zone from the disk. Then, the results were expressed as susceptible (S), intermediate (I), or resistant (R) as specified, following antibiotic discs instruction (BKMAM, Changde, China). All analyses were repeat three times.

### 2.3. Antibacterial Activity of L. sakei MS103 as Based on Inhibition of P. gingivalis Biofilm Formation

*P. gingivalis* cultures were adjusted to 6 Log CFU/mL with BHI broth after being incubated (37 °C) for 48 h. After *L. sakei* MS103 was cultured for 16 h, the strains with medium were collected directly without centrifuging to form the culture medium group. Next, the 2 transwell 24-well plates (0.4 μm, Corning, NY, USA) were used for culturing the *L. sakei* MS103 and *P. gingivalis* cells. The *L. sakei* MS103 included the culture group and supernatant group. The *L. sakei* MS103 and *P. gingivalis* cells of 1:1 (*v*/*v*) were added to the transwell plate. The *L. sakei* MS103 cells were added into the upper chambers, while the *P. gingivalis* cells were added into lower chambers. After the transwell plates were incubated for 48 h, culture supernatants within lower chambers were removed and discarded gently. Next, PBS was gently pipetted into the bottom chambers, which were then gently aspirated to remove planktonic *P. gingivalis* cells from the biofilm at the bottom of each chamber. Thereafter, in the lower chamber, 2.5% (*v*/*v*) glutaraldehyde solution was used to fix the *P. gingivalis* biofilm at 4 °C overnight. Then, the chamber was dried at room temperature. Next, staining at room temperature in each lower transwell chamber proceeded by using 100 μL of 0.1% (*w*/*v*) crystal violet for 15 min (room temperature). Afterwards, 75% ethanol was used to remove the unbound crystal violet of the stained biofilm. Then, the chamber was allowed to dry at room temperature [32].

A phase contrast fluorescence microscope (IX73, Olympus, Kyoto, Japan) was used to observe the *P. gingivalis* biofilm structure. A microplate reader was used to measure the biofilm absorbance at 540 nm. The biofilm inhibition rate of *L. sakei* MS103 to *P. gingivalis* was calculated as:

$$\mathrm{Biofilm}\;\mathrm{inhibition}\;\mathrm{rate}\;(\%)=\left[1-A_{s}/A_{c}\right]\times 100$$

where *A_S_* and *A_C_* represent the OD_540_ value of the *P. gingivalis* biofilm in the absence and presence of treated *L. sakei* MS103, respectively.

### 2.4. Antioxidant Activity In Vitro

Live and heat-killed *L. sakei* MS103 were prepared as described in Section 2.1. The antioxidant activities of 1 × 10^8^ CFU/mL live and heat-killed *L. sakei* MS103 in culture medium were measured for all groups. For the culture medium group, the *L. sakei* MS103 strains were collected with medium to adjust the concentration without centrifuge.

#### 2.4.1. DPPH Free Radical-Scavenging Activity (RSA)

A DPPH Free Radical Scavenging Capacity Assay Kit (BC4750, Solarbio, Beijing, China) was used to measure the scavenging of DPPH radicals. *L. sakei* MS103 was cultured, collected, and then washed with PBS at pH 7.0 twice. The *L. sakei* MS103 concentration was adjusted to 1 × 10^8^ CFU/mL, and then *L. sakei* MS103 was mixed with 100% ethanolic DPPH solution (0.2 mM) in a 1:2 ratio (*v*/*v*). After the mixture was incubated at 25 °C in the dark for 30 min, it was centrifuged for 10 min at 6000× *g*. The same volume of deionized water was added in the control group instead of the bacterial sample. For the blank group, the DPPH radical solution were replaced by ethanol with the same volume. The absorbance at 517 nm was measured.

DPPH-free RSA was calculated as follows:

$$\mathrm{DPPH-RSA}\;(\%)=\left[1-\frac{\left(A_{s}-A_{c}\right)\;}{A_{b}}\right]\times 100$$

where *A_s_*, *A_c_* and *A_b_* denote the absorbance of the test sample, control group and blank group, respectively.

#### 2.4.2. ABTS Radical Scavenging Assay (ABTS-RSA)

The decreasing of absorbance at 405 nm shows the ability of an antioxidant to scavenge the ABTS radical cation (ABTS^•+^). The ABTS radical-scavenging activity was assessed using an ABTS Radical Scavenging Assay Kit (BC4770, Solarbio).

The probiotic ABTS-RSA (%) was calculated as:

$$\mathrm{ABTS-RSA}\;(\%)=\left[1-\frac{\left(A_{s}-A_{c}\;\right)}{A_{b}}\right]\times 100$$

where *A_s_*, *A_c_* and *A_b_* denote the absorbance of the test sample, control group and blank group, respectively.

#### 2.4.3. Hydroxyl Radical Scavenging Assay

The hydroxyl free radical-scavenging activity was measured using a Hydroxyl Free Radical Scavenging Capacity Assay Kit (BC1329, BC4770, Solarbio). Briefly, the sample suspension (0.5 mL) was mixed with PBS (1 mL), phenanthroline (2.5 mL, 2.5 mM), FeSO_4_ (0.5 mL, 2.5 mM), and H_2_O_2_ (0.5 mL, 2.5 mM). Then, the mixture was incubated for 1 h with the temperature at 37 °C. Then, the mixture was centrifugated for 10 min (6000× *g*), and the absorbance at 536 nm was observed.

The hydroxyl free radical-scavenging activity was calculated as follows:

$$\mathrm{Scavenging}\;\mathrm{activity}\;(\%)=[\frac{\left(A_{s}-A_{c}\right)}{A_{b}-A_{c}}]\times 100$$

where *A_s_*, *A_c_* and *A_b_* denote the absorbance of the test sample, the control group without the sample and the blank group (without the sample and H_2_O_2_), respectively.

#### 2.4.4. Superoxide Anion Radical Scavenging Assay

The superoxide anion radical scavenging assay was conducted using a previously reported method [33,34]. First, 3.0 mL of Tris-HCl solution at pH 8.2 was mixed with 1.0 mL of sample and incubated for 20 min under 25 °C. Next, the mixture was incubated for 4 min (room temperature) after 0.4 mL of pyrogallol (25 mM) was added. Thereafter, to stop the reaction, HCl (0.5 mL) was added. The absorbance at 325 nm was observed. The control group contained an equivalent volume of deionized water instead of sample. The superoxide anion radical scavenging of the *L. sakei* MS103 was calculated as:

$$\mathrm{Scavenging}\;\mathrm{activity}\;\%=\left[\left(A_{c}-A_{s}\right)/A_{c}\right]\times 100$$

where *A_S_* and *A_C_* denote the absorbance at 325 nm for the test sample and control, respectively.

#### 2.4.5. Ferrous Ion-Chelating (FIC) Assay

First, 1 mL of *L. sakei* MS103 suspensions at different cell concentrations was mixed with FeCl_2_ solution (0.05 mL, 2 mM) for 5 min. Next, phenanthroline (0.2 mL, 5 mM) was added to each sample. Thereafter, 2.75 mL of distilled water was added to each sample; then, each sample was gently mixed for 10 min. Next, each sample was centrifuged at 2300× *g* for 10 min at 4 °C; then, the absorbances of samples were measured at 562 nm. For the control group, the sample (1 mL) and distilled water (3 mL) was mixed. For the blank group, 1 mL of water (instead of sample) was mixed with 0.05 mL FeCl_2_ (2 mM) solution, 0.2 mL of phenanthroline (5 mM) and 2.75 mL of distilled water. The ferrous ion-chelating (FIC) ability was calculated as:

$$\mathrm{Ferrous}\;\mathrm{Ion}\;\mathrm{Chelating}\;(\mathrm{FIC})\;(\%)=\left[1-\frac{\left(A_{s}-A_{c}\right)}{A_{b}}\;\right]\times 100$$

where *A_s_*, *A_c_* and *A_b_* denote the absorbance of the test sample, the control group and the blank group, respectively.

### 2.5. Antioxidant Properties of L. sakei MS103 as Assessed Using the H_2_O_2_-Induced Oxidative Damage RAW 264.7 Cell Model

#### 2.5.1. Cytotoxicity Text

As a reported method, a Cell Counting Kit-8 (CCK-8) assay (APExBIO, Houston, TX, USA) was used to observed the cytotoxicity of *L. sakei* MS103 against RAW 264.7 cells [35]. Briefly, 200 µL of a cell suspension of RAW 264.7 cells at a density of 5 × 10^4^/mL was added to individual wells of 96-well plates. After the adherent RAW 264.7 cells confluence reached 70–80% at 1 × 10^6^ cells/well, the live or heat-killed *L. sakei* MS103 cells at 0 Log CFU/mL, 7 Log CFU/mL, 8 Log CFU/mL, and 9 Log CFU/mL concentration were added to wells independently. After incubating the plate for 6 h, 300 μL of CCK-8 working reagent was added after the culture medium was removed. The multi-function microplate reader (F200 Pro, Tecan Infinite, Männedorf, Switzerland) was used to observe the OD identity at 450 nm for each well after the plates had been incubating for 4 h.

Cell viability was calculated as:

$$\mathrm{Cell}\;\mathrm{viability}\;(\%)=[A_{s}/A_{c}]\times 100$$

where *A_S_* and *A_C_* denote the OD_450_ absorbance of RAW264.7 cells co-incubated with *L. sakei* MS103 cells and without *L. sakei* MS103 cells, respectively.

#### 2.5.2. Assays for Measuring Glutathione (GSH) and Malondialdehyde (MDA) Levels

First, 5 × 10^4^ cells were added to individual wells of 96-well plates in 200 µL of medium. Then, the plates were incubated under standard culture conditions until the cells grew to 80–90% confluence. Next, supernatants were discarded, using PBS to wash the cells; then, 200 μL of DMEM containing 0.1 mmol/L H_2_O_2_ was added to wells to generate the H_2_O_2_-Induced Oxidative Damage RAW 264.7 Cell Model. Thereafter, live or heat-killed *L. sakei* MS103 cells were co-incubated with H_2_O_2_-exposed RAW 264.7 cells for 6 h. Concurrently, controls that included the blank (DMEM alone) and H_2_O_2_-damage positive control (DMEM containing 0.1 mmol/L H_2_O_2_) groups were incubated in the same way as the sample group. Concentrations of GSH and MDA were selected according to the instructions provided with the GSH assay kit (BC1175, Solarbio, Beijing, China) and MDA assay kit (BC0020, Solarbio). For GSH and MDA, the absorbance was measured at 412 nm and 532 nm with a spectrometer, respectively.

### 2.6. RNA Extraction and gshR4, Gpx, npx, and gshR2 Gene Expression

After RAW 264.7 cells were co-cultured with either live *L. sakei* MS103 or heat-killed *L. sakei* MS103 in medium including 0.l mM H_2_O_2_, treated RAW 264.7 cells were transferred to centrifuged tubes for RNA extraction. The total RNA was extracted from cells with the RNA Extraction Kit (TaKaRa 9767, Kyoto, Japan). The reverse transcription of total RNA (approximately 0.5 μg) into cDNA was completed by the Primescript™ RT reagent Kit (TaKaRa) [36,37]. The RNA subjected for reverse transcription are listed in Table S2. The relative transcriptional-level expression of *gshR4*, *Gpx*, *npx*, and *gshR2* were determined by cDNA, which served as a template. Real-time -PCR was performed by a TB Green^®^ Premix Ex Taq™ II Kit (TaKaRa, RR820A). The *β-actin* were served and normalized as the reference gene. The qRT-PCR results were calculated by the 2^−ΔΔCt^ method. The specificity of the reaction was evaluated by the melting curve analysis. In Table 1, the primer sequences are listed.

### 2.7. Statistical Analysis

Statistical calculations and computational data analysis were performed using GraphPad Prism 8.0 software (GraphPad Software Inc., San Diego, CA, USA). All data are expressed as the mean ± SD. Comparisons between groups were conducted using *t*-tests. One-way Analysis of Variance (ANOVA) was conducted to assess the statistical significance of differences in results obtained between groups receiving different treatments [38]. Values of *p* < 0.05 were considered statistically significant.

## 3. Results and Discussion

### 3.1. Characterization of L. sakei MS103

Probiotic bacterial strains play a crucial role in promoting health primarily by exerting clinically relevant effects within the gastrointestinal tract. To attain probiotic status, these strains must tolerate extreme conditions while passing through the stomach and other harsh environments within the human body.

**Figure 1:**
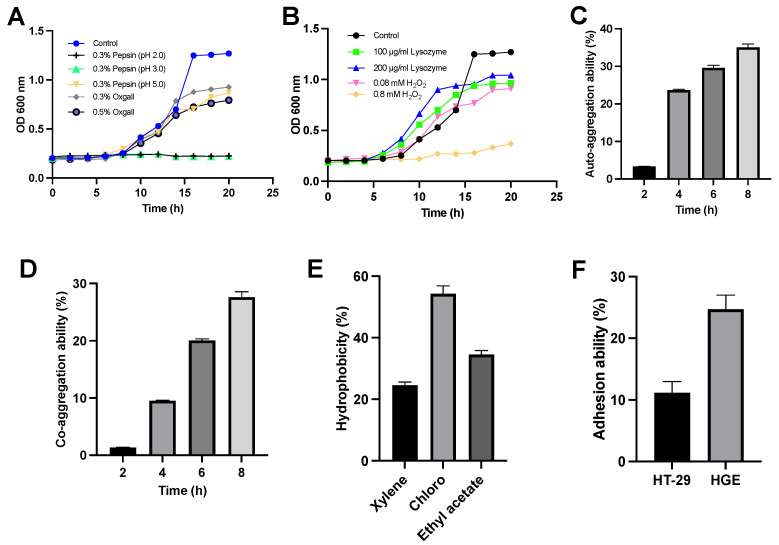
Characterization of *L. sakei* MS103 (**A**): growth curves of *L. sakei* MS103 exposed to different MRS broth for 20 h. The control group was only MRS broth. The MRS broth contained 0.3% pepsin at pH 2.0, pH 3.0, pH 5.0, 0.3% oxgall and 0.5% oxgall, individually. (**B**) Growth curves of *L. sakei* MS103 at different MRS broth for 20 h. The MRS broth contained 100 μg/mL lysozyme, 200 μg/mL lysozyme, 0.08 mM H_2_O_2_ and 0.8 mM H_2_O_2_, individually. (**C**) The *L. sakei* MS103 auto-aggregation ability. (**D**) The *L. sakei* MS103 co-aggregation ability. (**E**) The *L. sakei* MS103 surface hydrophobicity. (**F**) The *L. sakei* MS103 adhesion ability to HT-29 and HE cells. Values represent the mean ± SEM (n = 3).

In Figure 1A, we present the results of experiments conducted to assess *L. sakei* MS103 survival and proliferation during exposure to 0.3% pepsin at pH < 5.0. The results demonstrate that *L. sakei* MS103 survived and showed continued growth with these conditions. Similarly, when cultured in bile acids (0.3% and 0.5% ox gall, *w*/*v*), *L. sakei* MS103 maintained high growth activity throughout the 20-h period. However, when exposed to 0.3% pepsin at pH 2.0 or 3.0, the *L. sakei* MS103 exhibited slower growth and only survived for approximately 10 h.

When exposed to H_2_O_2_ or lysozyme, *L. sakei* MS103 cells demonstrated tolerance to 0.08 mM H_2_O_2_ and 200 g/mL lysozyme, maintaining steady growth throughout the 20 h period of exposure (Figure 1B). However, when exposed to 0.8 mM H_2_O_2_ for 20 h, *L. sakei* MS103 cells exhibited slower growth. However, surviving *L. sakei* MS103 cells were detected, indicating some degree of tolerance to H_2_O_2_, which is a weak oxidant known to cause oxidative damage. This result suggests that *L. sakei* MS103 possesses antioxidant abilities, which is a trait observed in other probiotics as well. For instance, studies by Xie et al. observed strong *Lactiplantibacillus plantarum* GXL94 resistance to H_2_O_2_, indicating antioxidant activity [39]. Similarly, research by Angmo’s group and Chul’s group revealed that certain LAB strains isolated from fermented foods survived exposure to low pH, bile salts, and lysozyme [40,41]. Based on these results, we speculate that *L. sakei* MS103 possesses antioxidant activity, enabling this strain to survive extreme environmental conditions.

Importantly, the auto-aggregate tendency was closely related to the adherence to the host cells of LAB. Here, *L. sakei* MS103 cells exhibited a 3.3% auto-aggregation rate in the first 2 h, which increased to 35% after 8 h (Figure 1C). When *L. sakei* MS103 was mixed with *P. gingivalis*, its co-aggregation rate at 2 h was low (1.3%); then, it gradually rose and reached a peak of 27.6% at 8 h (Figure 1D), indicating the agglutination of *L. sakei* MS103 with *P. gingivalis*. Notably, LAB strains with strong auto-aggregation abilities can efficiently colonize mucosal surfaces, enhancing their probiotic functions relative to those of strains lacking this ability [42]. Additionally, probiotics with co-aggregation abilities can produce antimicrobial substances that can inhibit activities near targeted organisms [40].

In this study, *P. gingivalis* served as a model pathogen that was used to assess *L. sakei* MS103 cells’ co-aggregation ability. Importantly, there was a progressive enhancement in *L. sakei* MS103 cells’ auto-aggregation capability with increasing incubation time along with robust *L. sakei* MS103 cells’ co-aggregation capability when co-incubated with *P. gingivalis*, underscoring the potential of this probiotic to serve as a treatment for combating bacterial infections, warranting further study.

Cell surface hydrophobicity is one of several key factors involved in non-specific cellular adhesion [43]. Bacterial colonization, which reflects cell surface hydrophobicity, is influenced by interactions between LAB and the environment or host. The results obtained in our study (Figure 1E) revealed a hydrophobicity value for *L. sakei* MS103 in xylene of 24.57%, indicating significant hydrophobicity. Changes in the *L. sakei* MS103 surface hydrophobicity were noted when it was exposed to acidic solvent (chloroform) and alkaline solvent (ethyl acetate), as reflected by probiotic hydrophobicity rates in chloroform and ethyl acetate of 54.31% and 34.53%, respectively. These results suggest the presence of numerous charges on *L. sakei* MS103 cell surfaces that exert electrostatic forces. Importantly, *L. sakei* MS103 exhibited a higher affinity for chloroform than for ethyl acetate, which was consistent with the published results for other LAB and *Latilactobacillus* strains [44,45].

Adhesion is a crucial property that shields probiotics from immediate elimination, granting them a competitive edge [31,46]. In this study, we found that an adhesion rate of *L. sakei* MS103 was 11.15% to HT-29 cells and 24.7% to HGE cells. This demonstrates that *L. sakei* MS103 has a higher adhesion ability to HGE cells compared to HT-29 cells. Pathogenic bacteria and viruses often rely on adhesion to host tissues to exert their pathogenic effects [47]. Therefore, *Lactiplantibacillus* strains with strong adhesion abilities to the same tissues may inhibit pathogen invasion and host cell-binding activities effectively [48,49]. Based on our results (Figure 1F), *L. sakei* MS103 shows promise as a potential treatment for oral diseases caused by bacterial strains with high adhesion to HGE cells.

Before combining antibiotic therapy with a probiotic, it is essential to assess the probiotic’s antibiotic sensitivity. This ensures that antibiotics do not inactivate the probiotic organisms and that antibiotic genes within the probiotic are not horizontally transferred to other bacterial species or disseminated to the environment. Therefore, the antibiotic sensitivity of a probiotic is a crucial factor that should be considered before administering it to patients.

In this study, the agar diffusion method was used to assess the sensitivity of *L. sakei* MS103 to 18 antibiotics following the Clinical and Laboratory Standards Institute (CLSI) guidelines (2020) [50]. The results in Table 2 indicate that *L. sakei* MS103 is sensitive to minocycline, gentamicin, polymyxin B, ceftazidime, and vancomycin, resistant to tetracycline, piperacillin, ampicillin, chloramphenicol, rifampicin, cefazolin, ceftazidime, and ceftriaxone, and moderately sensitive to erythromycin, doxycycline, streptomycin, and cefoperazone.

Notably, Özden et al. reported that a probiotic *L. sakei* strain isolated from fermented Turkish sausage exhibited sensitivity to vancomycin and gentamicin, which was consistent with our results for *L. sakei* MS103 [51]. Other research groups have reported that numerous *Limosilactobacillus* and *Lactiplantibacillus* strains are resistant to ceftriaxone and cefazolin, which is in alignment with our findings for *L. sakei* MS103 [52]. While strains from different sources may possess varying antibiotic susceptibility profiles, our results suggest that *L. sakei* MS103 exhibits suitable antibiotic sensitivity for use as a probiotic.

### 3.2. Antibacterial Activity of L. sakei MS103 against P. gingivalis Biofilm Formation

*P. gingivalis* is a key pathogen that participates in the formation of mature dental plaque. It can form biofilms along with various pathogenic and non-pathogenic bacterial species, contributing to the development of periodontal disease. Investigating biofilm formation by *P. gingivalis* in the presence of a probiotic can provide valuable insights for preventing periodontitis.

**Figure 2:**
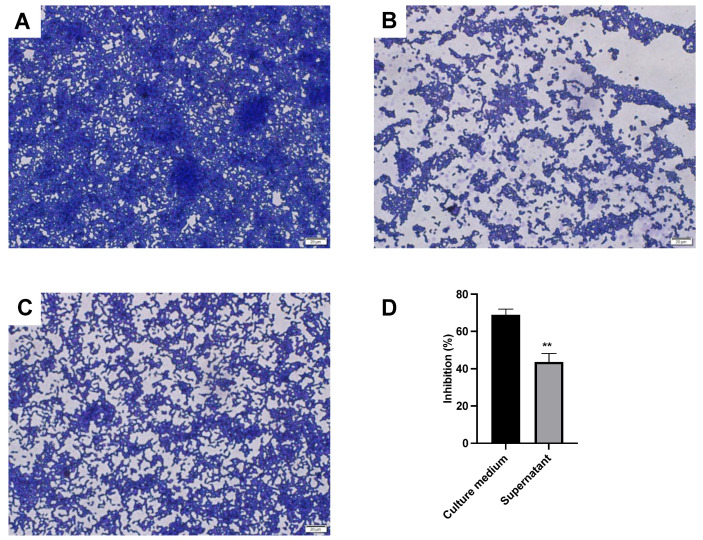
The *L. sakei* MS103-treated *P. gingivalis* biofilm. After being cultured for 48 h, the *P. gingivalis* biofilm was stained with crystal violet. (**A**) Control group without *L. sakei* MS103. (**B**) *L. sakei* MS103 culture medium treated for *P. gingivalis*. (**C**) *L. sakei* MS103 supernatant treated for *P. gingivalis*. (**D**) Inhibition rate of *L. sakei* MS103 on biofilm formation of *P. gingivalis*. Values represent the mean ± SEM (n = 3). ** *p* < 0.01.

In our experiments, we observed crystal violet-stained *P. gingivalis* biofilms under a microscope. Highly structured and irregular biofilm structures containing numerous *P. gingivalis* organisms were observed in the control group with *P. gingivalis* cultured 48 h along with several mushroom-like microcolonies (Figure 2A).

In contrast, *P. gingivalis* biofilm formation was significant inhibited by adding *L. sakei* MS103 culture medium, resulting in the formation of biofilm consisting of only a single layer of *P. gingivalis* cells with a loose structure lacking membranous characteristics (Figure 2B). When *P. gingivalis* was treated with cell-free *L. sakei* MS103 supernatant, which was an unevenly distributed biofilm formed that contained large gaps in its structure and loosely arranged *P. gingivalis* cells accompanied by smaller mushroom-shaped microcolonies (Figure 2C). Furthermore, the addition of *L. sakei* MS103 culture medium or bacteria-free culture supernatant to *P. gingivalis* cultures inhibited biofilm formation by 65.29% and 38.62%, respectively (Figure 2D). In culture medium groups, after *L. sakei* MS103 was cultured for 16 h, the strains with medium were collected directly without centrifuging. These results indicate that the *L. sakei* MS103 culture medium significantly outperformed cell-free *L. sakei* MS103 supernatant in inhibiting *P. gingivalis* biofilm formation.

Numerous probiotics have been reported to interfere with biofilm formation by oral pathogens, aligning with our findings. Consequently, the screening of probiotic candidates for capabilities in inhibiting activities of human oral bacteria may be a valuable approach for preventing dental caries and other oral diseases.

Notably, *Lactiplantibacillus* strains have been reported to effectively prevent biofilm formation by *Salmonella* spp. [53]. Additionally, lipoteichoic acids produced by *lactobacilli* have been shown to inhibit *E. faecalis* biofilm formation and disrupt preformed biofilm structures [54,55]. Therefore, with inhibiting the biofilm formation of *P. gingivalis*, the *L. sakei* MS103 provides an advantage for making some oral health products in the future. Our results imply that *L. sakei* MS103 possesses probiotic characteristics that may enable it to inhibit biofilm formation by pathogenic bacteria.

### 3.3. Results of In Vitro Assays of Antioxidant Activity Induced by L. sakei MS103 Treatment

**Figure 3:**
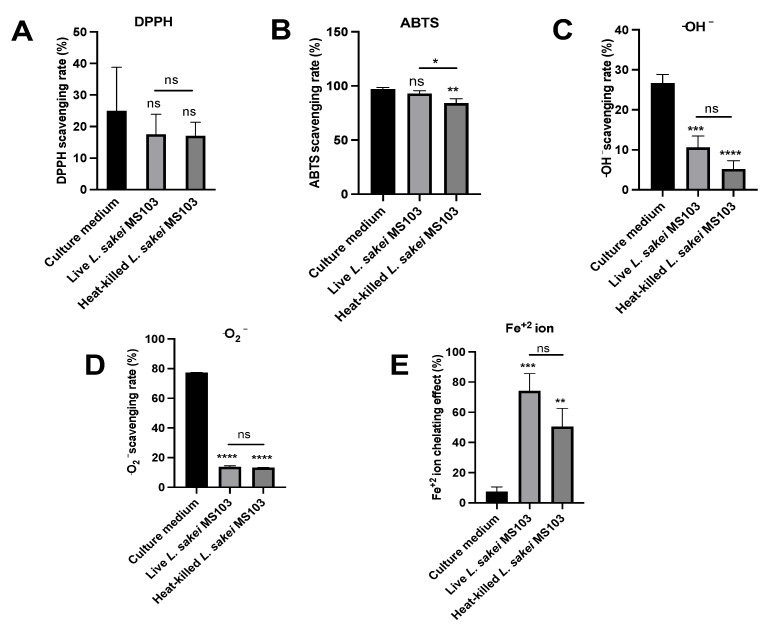
Antioxidant activity induced by *L. sakei* MS103 treatment in culture medium, live *L. sakei* MS103 and heat-killed *L. sakei* MS103. (**A**) DPPH-free RSA. (**B**) ABTS-RSA. (**C**) Hydroxyl radical RSA. (**D**) Superoxide anion scavenging activities. (**E**) Fe^2+^-chelating capacity. Values represent the mean ± SEM (n = 3). * *p* < 0.05, ** *p* < 0.01, *** *p* < 0.001, **** *p* < 0.0001.

In biological systems, DNA can be damaged by ROS such as superoxide and hydroxyl radicals. To mitigate these effects, probiotics with antioxidative activities have been developed to safeguard cells from oxidative damage. This study assesses *L. sakei* MS103 antioxidative activities, including ferrous ion-chelating (FIC) ability and *L. sakei* MS103 activities related to the scavenging of the superoxide anion, DPPH, hydroxyl radical free radicals, and ABTS, by comparing activities of *L. sakei* MS103 culture medium, live *L. sakei* MS103, and heat-killed *L. sakei* MS103 to controls (Figure 3).

DPPH radical scavenging assays are commonly employed to assess antioxidant activities that are primarily related to hydrogen-donating capabilities. In Figure 3A, it is evident that the live *L. sakei* MS103 group (39.15%) exhibited higher DPPH radical-scavenging activity as compared that of the *L. sakei* MS103 culture medium group (24.96%) and heat-killed *L. sakei* MS103 group (17.01%).

**Figure 4:**
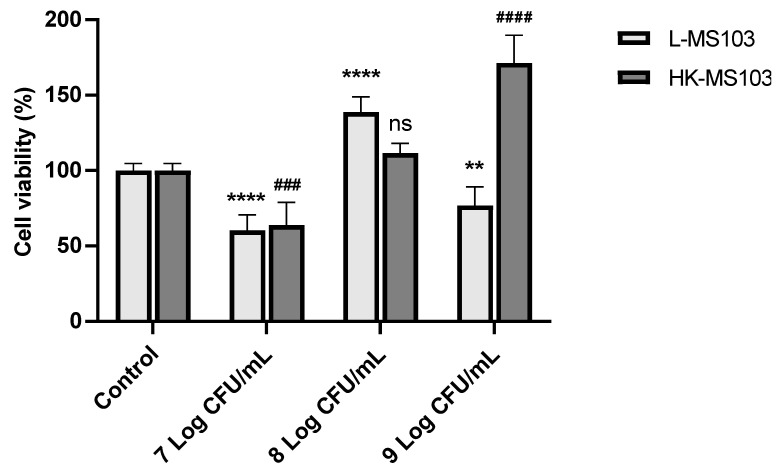
Effect of *L. sakei* MS103 on the cell viability of RAW264.7 cells. Values represent the mean ± SEM (n = 3). ** *p* < 0.01, **** *p* < 0.0001, live *L. sakei* MS103 compared to control group; ^###^ *p* < 0.001, ^####^ *p* <0.0001, heat-killed *L. sakei* MS103 compared to control group.

In contrast, when assessing the total antioxidant capacities based on ABTS radical-scavenging ability, all three groups, *L. sakei* MS103 culture medium (97.16%), live *L. sakei* MS103 (93.06%), and heat-killed *L. sakei* MS103 (87.39%), exhibited excellent ABTS radical-scavenging abilities (Figure 4). These results collectively indicate that *L. sakei* MS103 possesses robust antioxidant capacity that is comparable to that reported for *L. sakei* KU15041 and *Latilactobacillus curvatus* KU15003 [56].

Primary ROS generated during cellular metabolism typically include superoxide anion (•O_2_^−^) and hydroxyl radical (•OH), among other types of free radicals. Among these, (•OH) poses a substantial threat to biological macromolecules, resulting in adverse effects on cell health. Therefore, (•OH)-scavenging capacity, an indicator of antioxidant activity, is a crucial factor to consider when selecting probiotics with resistance to ROS-induced damage.

In this study, we assessed the (•OH)-scavenging capacities of the *L. sakei* MS103 culture medium, live *L. sakei* MS103, and heat-killed *L. sakei* MS103 (Figure 3C). Our findings demonstrated that the *L. sakei* MS103 culture medium exhibited the highest (•OH)-scavenging capacity (26.69%), which was followed by live *L. sakei* MS103 cells (10.62%). Heat-killed *L. sakei* MS103 cells displayed the lowest overall (•OH) scavenging capacity (5.20%). Notably, these findings are consistent with results reported for other LAB strains showing that both live and heat-killed LAB possess (•OH)-scavenging capacity [57].

Another significant ROS, superoxide anion (•O_2_^−^), can lead to oxidative stress and biomolecular damage and thus was included in our analysis of *L. sakei* MS103 superoxide anion-scavenging abilities. Our findings revealed that both live and heat-killed *L. sakei* MS103 had similar (•O_2_^−^)-scavenging capacities of 13.77% and 13.27%, respectively (Figure 3D). Of note, the *L. sakei* MS103 culture medium exhibited significantly higher (•O_2_^−^)-scavenging ability (77.26%). In summary, our results demonstrate that *L. sakei* MS103 possesses remarkable superoxide anion scavenging capacity that is likely related to substances present on *L. sakei* MS103 cell surfaces.

Metal-chelating assays, particularly those used to assess Fe^2+^-chelating capacity [58], are valuable for understanding antioxidant mechanisms and mitigating Fenton reaction-induced free radical formation. Notably, Fe^2+^ chelation is an effective strategy for protecting cells from oxidative damage [59]. In this study, we found that live *L. sakei* MS103 exhibited an Fe^2+^-chelating capacity of 74.11%, while heat-killed *L. sakei* MS103 had a slightly lower capacity of 50.46%. Notably, the *L. sakei* MS103 culture medium displayed the lowest Fe^2+^-chelating capacity (7.57% (Figure 3E)). Previous studies have suggested that the strong (•OH)-scavenging activities of LAB strains may be linked to their abilities to bind to metal ions such as Fe^2+^ [60,61]. Additionally, some LAB species have shown antioxidant activities associated with the elimination of transition metal ions [62,63]. In summary, our results demonstrate the significant Fe^2+^-chelating capacity of *L. sakei* MS103, suggesting it may exert antioxidant effects by inhibiting metal ion-catalyzed free radical generation.

From our results, the *L. sakei* MS103 existed antioxidative activities but not significant differences between live and heat-killed *L. sakei* MS103 in the scavenging of DPPH, ABTS, superoxide anion, and hydroxyl radical free radicals and FIC ability.

### 3.4. RAW 264.7 Cell-Protective L. sakei MS103 Antioxidant Properties

#### 3.4.1. Effect of *L. sakei* MS103 on RAW 264.7 Cell Viability and Proliferation

Macrophages are important immune system cells that phagocytize pathogenic microorganisms [64]. However, the presence of bacteria and viruses can lead to increased macrophage ROS production that could lead to macrophage apoptosis when ROS levels become elevated [65,66,67].

The cytotoxic effects of *L. sakei* MS103 on RAW 264.7 cells were assessed. We examined RAW 264.7 cell viability following exposure to varying concentrations of *L. sakei* MS103. As shown in Figure 4, as compared to the control group, the addition of 7 Log CFU/mL live or heat-killed *L. sakei* MS103 led to reduced RAW 264.7 cell viability to only 60.4% and 63.98%, respectively. Intriguingly, at an *L. sakei* MS103 concentration of 8 Log CFU/mL, the cell viability increased significantly to 138.75%, which is a viability rate that was considerably higher than that of the control group. Similarly, RAW 264.7 cell viability increased after the addition of 8 Log CFU/mL heat-killed *L. sakei* MS103 (111.54%).

In contrast, 9 Log CFU/mL of live *L. sakei* MS103 added to RAW 264.7 cells led to decreased viability and the proliferation of RAW 264.7 cells, although no such inhibition was observed after the addition of 9 Log CFU/mL heat-killed organisms. These results indicate that both 7 Log CFU/mL of live or heat-killed *L. sakei* MS103 and 9 Log CFU/mL of live *L. sakei* MS103 exerted cytotoxic effects against RAW 264.7 cells, resulting in partial cell death and reduced RAW 264.7 cell viability. Therefore, the live or heat-killed *L. sakei* MS103 at 8 Log CFU/mL was used in further experiments.

#### 3.4.2. *L. sakei* MS103 Antioxidant Effects on RAW 264.7 Cell GSH and MDA Levels

H_2_O_2_ plays a pivotal role in the generation of highly diffusible and long-lasting active oxygen molecules, such as hydroxyl radicals, which have the potential to induce cellular oxidative damage through specific interactions [68]. Excessive exogenous H_2_O_2_ disrupts cellular equilibrium, leading to ROS overproduction and oxidative damage.

While ROS can damage cells, macrophages utilize ROS to directly eliminate invading pathogens, although this defense mechanism can lead to macrophage injury and cell death. To assess the potential antioxidant effect of *L. sakei* MS103, we employed the widely used H_2_O_2_-Induced Oxidative Damage RAW 264.7 Cell Model. This model measures cellular responses to oxidative stress based on intracellular GSH and MDA levels, whereby elevated GSH and reduced MDA levels indicate increased cellular antioxidant activity that can protect cells from H_2_O_2_-induced damage.

**Figure 5:**
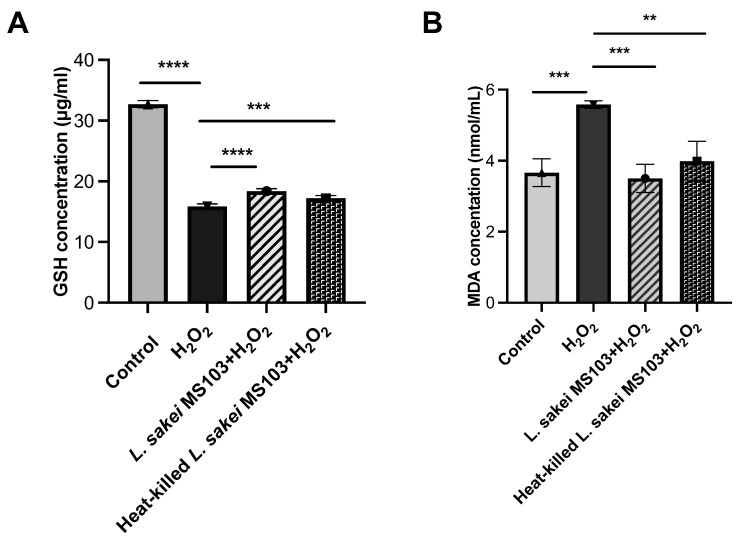
Concentration of GSH and MDA for RAW 264.7 cells after treatment with live and heat-killed *L. sakei* MS103. RAW264.7 cells were treated with live *L. sakei* MS103 or heat-killed *L. sakei* MS103 and effect with H_2_O_2_ (0.1 mmol/L) for 6 h. (**A**): GSH concentration changing results. (**B**): MDA concentration changing results. Values represent the mean ± SEM (n = 3). ** *p* < 0.01, *** *p* < 0.001, **** *p* < 0.0001, compared to the control group.

GSH concentrations in the following groups were observed: control (32.71 μg/mL), H_2_O_2_-exposed (15.89 μg/mL), live *L. sakei* MS103-treated H_2_O_2_-exposed (18.4 μg/mL), and heat-killed *L. sakei* MS103-treated H_2_O_2_-exposed (17.2 μg/mL) cells. The results indicate that the treatment of H_2_O_2_-exposed RAW 264.7 cells with either live or heat-killed *L. sakei* MS103 cells resulted in decreased intracellular GSH concentration (Figure 5A).

Furthermore, the treatment of H_2_O_2_-exposed RAW 264.7 cells with either live or heat-killed *L. sakei* MS103 cells led to increased intracellular MDA concentration as compared to that of the control group. These findings collectively demonstrate that both live and heat-killed *L. sakei* MS103 exhibit antioxidant capabilities that may reduce the H_2_O_2_-induced oxidative damage of macrophages (Figure 5B).

### 3.5. Gene Expression Changes in H_2_O_2_-Exposed RAW 264.7 Cells after L. sakei MS103 Treatment

**Figure 6:**
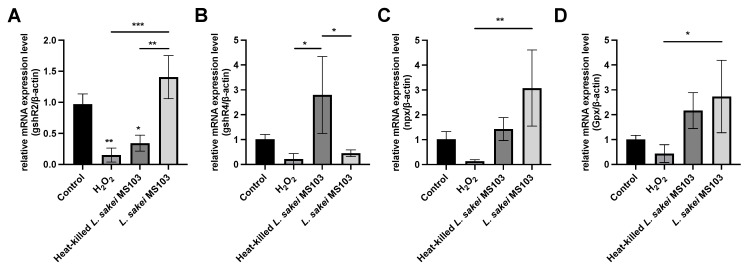
The mRNA expression for the H_2_O_2_ effect of RAW 264.7 cells treated with *L. sakei* MS103. Live or heat-killed *L. sakei* MS103 treated with RAW264.7 cells (effect with 0.1 mmol/L H_2_O_2_) for 6 h. The *β-actin* gene used to determine the mRNA levels of (**A**): *gshR2*, (**B**): *gshR4*, (**C**): *npx* and (**D**): *Gpx*. Values represent the mean ± SEM (n = 3). Comparison between H_2_O_2_ effect group and *L. sakei* MS103 treatment. * *p* < 0.05, ** *p* < 0.01, *** *p* < 0.001.

The above-mentioned results demonstrate that *L. sakei* MS103 cells’ antioxidative effects can protect H_2_O_2_-exposed RAW 264.7 cells from oxidative damage. To investigate the transcription-level mechanisms responsible for observed *L. sakei* MS103-induced increases in RAW 264.7 cell antioxidant activity, we assessed changes in the expression of four antioxidant response-related genes (*gshR4*, *Gpx*, *npx*, and *gshR2*). From the published results, these four genes were all related to the antioxidant effects [33]. These experiments were conducted using H_2_O_2_-exposed RAW 264.7 cells as a cellular oxidative damage model with and without added *L. sakei* MS103. Table S1 provides the RT-qPCR amplification parameters, with the results presented in Figure 6. Our findings revealed that H_2_O_2_ exposure down-regulated the mRNA-level expression of all four genes in RAW 264.7 cells. In contrast, the treatment of RAW 264.7 cells with live or heat-killed *L. sakei* MS103 significantly increased the mRNA-level expression of all these genes.

Genes encoding one or two glutathione reductases, referred to as *gshR* genes, are crucial components of the oxidative stress response mechanisms in various probiotic organisms, including *Streptococcus thermophilus* and *E. faecalis* [69]. In our study, these genes were found to play pivotal roles in the oxidative stress response of RAW 264.7 cells. When H_2_O_2_-exposed RAW 264.7 cells were treated with either live or heat-killed *L. sakei* MS103, we observed 2.6-fold and 9.2-fold increases in *gshR2* expression, respectively (Figure 6A). In contrast, both treatments led to increased *gshR4* expression (0.44-fold and 2.7-fold up-regulation, respectively), indicating heat-killed *L. sakei* MS103 induced a greater increase in *gshR4* expression as compared that induced by live *L. sakei* MS103 (Figure 6B). Taken together, these results indicate that the addition of either live or heat-killed *L. sakei* MS103 to oxidatively stressed RAW 264.7 cells induced up-regulated RAW 264.7 cell expression of both *gshR2* and *gshR4* relative to controls. However, a greater up-regulation of *gshR4* expression was induced by treatment with heat-killed organisms, while live organisms induced a greater up-regulation of *gshR2* expression.

Furthermore, *npx* gene expression, which is associated with NADH oxidase-NADH peroxidase system function [70], showed significant up-regulation in model cells following treatment with either live *L. sakei* MS103 (23.9-fold) or heat-killed *L. sakei* MS103 (11.1-fold) (Figure 6C). These findings, along with the above-mentioned *gshR* results, strongly suggest that the *L. sakei* MS103 treatment-induced up-regulated expression of *gshR* and *npx* plays a crucial role in mitigating the oxidative damage of H_2_O_2_-exposed RAW 264.7 cells.

Similarly, treatment of the oxidative stress model with either live or heat-killed *L. sakei* MS103 induced an up-regulation of *Gpx* gene expression (2.7-fold and 2.1-fold increases, respectively (Figure 6D), thus underscoring the antioxidant potential of *L. sakei MS103* in reducing the effects of oxidative stress. Taken together, the above-mentioned results demonstrate that most of the key genes were up-regulated in *L. sakei* MS103-treated model cells. While further research is needed to fully understand the mechanisms underlying these antioxidant effects, our results support *L. sakei MS103* as a promising antioxidant probiotic for use in various applications.

## 4. Conclusions

In this study, we assessed the antioxidant potential of *L. sakei* MS103, which is a probiotic strain isolated from a traditional fermented food known as sweet pickled garlic. This remarkable biosafe strain exhibits robust survivability in harsh environmental conditions, including hydrogen peroxide, lysozyme exposure, low pH levels and bile salts. Additionally, it possesses the abilities of auto-aggregation, co-aggregation, and adhesion, facilitating its adhesion and colonization within specific microenvironments. Furthermore, our in vitro experiments demonstrated that *L. sakei* MS103 effectively inhibited *P. gingivalis*-induced biofilm formation. Additionally, *L. sakei* MS103 exhibited antioxidant properties with antioxidative activities. Notably, the addition of *L. sakei* MS103 to H_2_O_2_-exposed RAW 264.7 cells led to increased RAW 264.7 cell antioxidative activity, as reflected by the significant regulated expression of four RAW 264.7 cell genes associated with oxidative stress responses following *L. sakei* MS103 treatment. Collectively, these findings strongly suggest that *L. sakei* MS103 holds promise as a potent candidate treatment for conditions related to oxidative stress.

## Figures and Tables

**Table 1:** Primer sequences.

Gene	Primer Sequence (5′–3′)
*β-actin*	F: GTGGGCCGCCCTAGGCACCAG
	R: GGAGGAAGAGGATGCGGCAGT
*Gpx*	F: GCGAGCTCATGGCAGAATCAGTGTATGATTT
	R: CCCAAGCTTTTAATCTTCTGAACGATCAGCC
*gshR2*	F: GCGAGCTCATGTCAGAAAAATTTGACGTTGT
	R: CCCAAGCTTTTAAATTGCTGACCAAACGG
*gshR4*	F: GCGAGCTCATGACAAACAAATACGATTACGATGTG
	R: CCCAAGCTTTTAAGCCCGGTGCCAAGC
*npx*	F: GCGAGCTCATGGCAAAAATTATTATTGT
	R: CCCAAGCTTCTAGTTAGTGGCTAAAGTTTGT

**Table 2:** *L. sakei* MS103 antibiotic sensitive ability.

Antimicrobial Agent		Disk Content (μg)	Criteria of Inhibition Zone Diameters (mm)			Detection Result	
Group	Drug		R	I	S	Inhibition Zone (mm)	Sensibility *
Glycopeptis	Vancomycin	30	≤14	-	≥15	19.9	S
Lipopeptids	Polymyxin B	300	≤8	9–11	≥12	30.1	S
Ansamycins	Rifampicin	5	≤16	17–19	≥20	0	R
Chloramphenicol	Chloramphenicol	30	≤13	14–17	≥18	13.0	R
Cephems	Cefazolin	30	≤19	20–22	≥23	15.6	R
	Cefuroxime	30	≤14	15–22	≥23	0	R
	Ceftriaxone	30	≤19	20–22	≥23	0	R
	Cefoperzone	75	≤15	16–20	≥21	19.7	I
	Ceftazidime	30	≤17	18–20	≥21	21.9	S
Macrolides	Doxycycline	30	≤12	13–15	≥16	13.2	I
Tetracyclins	Erythromcin	15	≤15	16–20	≥21	16.4	I
	Minocyline	30	≤12	13–15	≥16	21.3	S
	Tetracycline	30	≤11	12–14	≥15	0	R
β-Lactams penicillins	Penicillin	10	≤28	-	≥29	18.5	R
	Piperacillin	100	≤17	18–20	≥21	12.7	R
	Ampicillin	10	≤11	12–14	≥15	0	R
Aminoglycosides	Streptomcin	10	≤12	13–14	≥15	13.8	I
	Gentamicin	10	≤12	13–14	≥15	26.4	S

* S—susceptible; I—intermediate; R—resistance.

## Data Availability

Data is contained within the article or Supplementary Material.

## Supplementary Materials

The following supporting information can be downloaded at https://www.mdpi.com/article/10.3390/foods12234276/s1, Table S1: Antioxidant ability of the lactic acid bacteria; Table S2: The RNA concentration and RNA concentration.
