# Supplementary material for: Antioxidant Effects and Probiotic Properties of Latilactobacillus sakei MS103 Isolated from Sweet Pickled Garlic
Source: Foods. 2023 Nov 27;12(23):4276. doi: 10.3390/foods12234276 (PMC10706130; doi:10.3390/foods12234276)
Supplement: Supplementary file 1 [file foods-12-04276-s001.zip › foods-2738026-supplementary.pdf]

*Article*

# Antioxidant Effects and Probiotic Properties of *Latilactobacillus sakei* MS103 Isolated from Sweet Pickled Garlic

**Heng Li [1,2], Changlin Chen[1], Yuanxin Li[2], Zhengqiang, Li [2], Chen Li[3] *and Chang Luan [2,4]***

1 College of Information Technology, Jilin Agricultural University,Chuangchun,130118, China; hengl@jlau.edu.cn (H.L) ; 20231321@mails.jlau.edu.cn (C.C)

2 Key Laboratory for Molecular Enzymology and Engineering of the Ministry of Education, College of Life Sciences, Jilin University, Changchun 130012, China; hengl@jlau.edu.cn (H.L) ; lzq@jlu.edu.cn (Z.L.); Yuanxin22@mails.jlu.edu.cn (Y.L); luanchang6789@163.com (Chang.Luan.)

3 State Key Laboratory for Zoonotic Diseases, Key Laboratory for Zoonosis Research of the Ministry of Education, Institute of Zoonosis, College of Veterinary Medicine, Jilin University, Changchun 130062, China;

4 State Key Laboratory of Black Soils Conservation and Utilization, Northeast Institute of Geography and Agroecology, Chinese Academy of Sciences, Changchun 130102, China, luanchang6789@163.com (Chang.Luan)

* Correspondence: lc2018@jlu.edu.cn (Chen.Li.); luanchang@iga.ac.cn (Chang.Luan);

## 1. Preliminary screening of antioxidant ability with DPPH and ABTS concentration.

The lactic acid bacteria cultured for after 16 h was centrifuged (1500 xg, 10 min at 4 ℃, the supernatant was collected. Using the 0.22 μm filtered to obtain the lactic acid bacteria supernatant. The DPPH and ABTS were observed by DPPH Free Radical Scavenging Capacity Assay Kit (BC4750, Solarbio, Beijing, China ) and ABTS Free Radical Scavenging Capacity Assay Kit (BC4770, Solarbio, Beijing, China ), respectively.

Table S1 Antioxidant ability of the lactic acid bacteria

| Strain | DPPH radical scavenging activity (%) | ABTS radical scavenging activity (%) |
|---|---|---|
| MS105 | 27.33 | 90.91 |
| AC1-2 | 27.7 | 97.67 |
| Lgg | 34.27 | 86.47 |
| LVBH107 | 31.41 | 95.77 |
| BSF210 | 36.36 | 80.34 |
| BSF206 | 31.76 | 90.95 |
| BSA103 | 26.40 | 88.37 |
| MS103 | 35.37 | 99.45 |
| TH103 | 27.16 | 14.80 |
| BSH510 | 30.44 | 71.25 |

## 2. Expression of genes related to biofilm synthesis

RNA was reverse transcribed into cDNA by Primescript™ RT reagent kit (Takara, Japan). The total amount of RNA in the reverse transcription system was 0.5 μg. The specific RNA dosage is shown in the table below.

Table S2 The RNA concentration and RNA volume

| Sample | RNA concentration ( ng/μL ) | RNA volume ( μL ) |
|---|---|---|
| Control | 148.5 | 3.7 |
| $H_2O_2$ | 222.8 | 2.2 |
| Heat-killed *L. sakei MS103* | 348.0 | 1.4 |
| *L. sakei MS103* | 80.0 | 6.3 |
